# Patient-reported Outcomes of Short-term Intra-articular Hyaluronic Acid for Osteoarthritis of the Knee: A Consecutive Case Series

**DOI:** 10.7759/cureus.4972

**Published:** 2019-06-22

**Authors:** Charles A Gusho, Mark Jenson

**Affiliations:** 1 Miscellaneous, Medical College of Wisconsin Green Bay, De Pere, USA; 2 Family Medicine, Medical College of Wisconsin Green Bay, De Pere, USA

**Keywords:** osteoarthritis, hyaluronic acid, viscosupplementation

## Abstract

Background

Supartz FX (Seikagaku Corp., Tokyo, Japan) has been investigated as a therapeutic for knee osteoarthritis (OA) due to its claimed preservation of viscoelastic joint properties and improvement in pain and physical function. The US prescribing information suggests patients may experience benefit with as few as three of five injections administered once weekly. However, recommended guidelines from the American Academy of Orthopaedic Surgeons (AAOS) do not support injectable hyaluronate due to controversial results of randomized controlled trials.

Objective

Do patients experience statistically and/or clinically significant improvement in disability scores following three injections?

Methods

A total of 32 patients with a mean age of 66±14 years receiving Supartz FX were reviewed in a prospective, observational study. Functional outcome data via Western Ontario and McMaster Universities Osteoarthritis Index (WOMAC) scores for pain, stiffness, and physical function were collected at 0, 1, 2 and 3 weeks, and means were analyzed via paired t-test.

Results

Three injections at one-week intervals resulted in statistically significant improvement across all sub scores (p<0.05). Confidence intervals (CIs) of treatment effects (ES, 95% CI) for pain (0.27, 95% CI 0.99, 1.26), stiffness (0.17, 95% CI 0.50, 0.67), and function (0.55, 95% CI 2.79, 3.35) were recorded and compared to published minimum clinically important improvement (MCII) thresholds.

Conclusion

Despite manufacturer recommendations, in this study short-term use of Supartz FX for knee OA does not meet clinically significant thresholds as the treatment effects for WOMAC sub scores fail to satisfy published MCII for pain (0.39), stiffness (0.39) and function (0.37). In light of these findings and in concordance with recommendations set forth by the AAOS, this study contributes to a preventative medicine database that encourages exploration of non-surgical and non-opiate modalities for the management of osteoarthritis.

## Introduction

Osteoarthritis (OA) is the most common joint disorder in the United States, affecting approximately 27 million Americans [1]. OA most commonly occurs in the knee, and one in two adults will develop symptoms of knee OA sometime in their lives [1]. Specifically, OA of the knee is due in part to a decreased viscosity of synovial fluid which normally acts as a cushion. A healthy joint is lubricated with 1-2 mL of synovial fluid containing 5 to 8 mg of hyaluronic acid (HA) [2]. In the arthritic knee, however, HA is diminished, reducing the viscoelastic properties of the joint and increasing the stress on the articular surface, causing erosion, bone spurs, and pain [2].

Less severe forms of knee OA are commonly managed with intra-articular HA (IAHA) injections, though meta-analyses of randomized controlled trials have failed to identify significant clinical changes in outcomes between IAHA and placebo [3]. Balazs and Denlinger were the first to suggest IAHA use for restoration of viscoelastic properties and improved functionality, and several compounds with differing molecular weight, preparation of purified sodium hyaluronate, and injection schedules have since been introduced into clinical practice [4-5]. One such viscosupplement, Supartz FX (Seikagaku Corp., Tokyo, Japan), is administered as a 25 mg/2.5 mL intra-articular injection once weekly for five weeks, for a total of five injections [6].

Previous studies have explored the efficacy of high-molecular-weight HA models using Likert-type Western Ontario and McMaster Universities Osteoarthritis Index (WOMAC) scores for pain and physical function [7-8]. Additional studies have suggested improvement in WOMAC scores using Supartz FX followed out to 1, 3, and 6 months, with significant mean reduction at 26 weeks, and an overall delayed time to total joint arthroplasty [8-9]. However, according to US prescribing information, patients using Supartz FX may experience benefit after a minimum of three doses given at weekly intervals [10]. Therefore, the aim of this study was to analyze self-reported weekly percent improvements in pain, stiffness, and function using WOMAC scores following three consecutive Supartz FX injections in patients with knee OA.

## Materials and methods

Study subjects

Fifty-five patients (female = 27) were screened to participate in this study. The inclusion criteria were unilateral knee OA (Kellgren-Lawrence Grade 2 or 3) confirmed by radiographs. The exclusion criteria were bilateral knee OA, previous IAHA, viscosupplementation, or corticosteroid injection into the knee less than three months from starting Supartz FX; concomitant knee disease, ligamentous instability on physical exam, and ongoing anticoagulant therapy.

Study design

As a consecutive case-series, patient-reported outcomes following Supartz FX were recorded using WOMAC scores before and one-week after each of three injections. WOMAC scores are based on five items related to pain (sub score: 0-20; 0 = minimum pain sub score; 20 = maximum pain sub score), 2 items related to stiffness (sub score 0-8; 0 = minimum stiffness sub score; 8 = maximum stiffness sub score), and 17 items related to physical activity (sub score 0-68; 0 = minimum physical activity sub score; 68 = maximum physical activity sub score). The summed score is normalized to a total out of 100.

Statistical analysis

WOMAC sub score data, computed to a normalized value of 100, and mean percent reductions, were recorded at weeks 0, 1, 2 and 3. Sub scores were analyzed using a paired 2-sample t-test. A value of p<0.05 was considered statistically significant and data are represented as the mean ± standard error (SE) of the mean. Weekly sub score changes were statistically analyzed for effect size at a 95% confidence interval and compared to published minimum clinically important improvement (MCII) thresholds.

## Results

**Figure 1:**
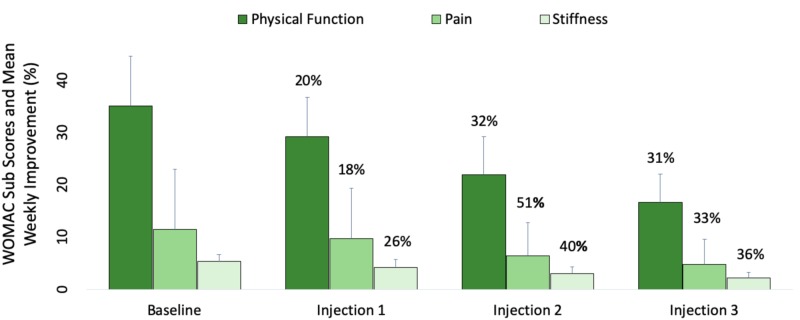
Effectiveness of Supartz FX injections into the knees of patients with osteoarthritis over a three-week period Thirty-two patients with a mean age of 66±14 years received intra-articular injections of Supartz FX once weekly for three consecutive weeks. Mean reduction in Western Ontario and McMaster Universities Osteoarthritis Index (WOMAC) scores for pain, stiffness and physical function were assessed at baseline and 1, 2, and 3 weeks while concurrently undergoing Supartz FX therapy. p<0.05 was used to statistically compare weekly mean values. n=32.

Thirty-two patients (females = 19) aged between 35-89 (66±14; mean ± SD) met the inclusion criteria. The average weight was 95.3±27.1 kg, and the average height was 1.67±0.11 m. The average body mass index (BMI) as 34.2±7.9 kg/m2. 23 patients were obese (BMI ≥ 30.00 kg/m2), seven were overweight (BMI 25.00-29.99 kg/m2), and two patients were considered normal weight (BMI 18.5-24.99 kg/m2). Percent mean improvement (%) in WOMAC sub scores were recorded weekly (Figure 1). 

A decrease from "Baseline" sub scores for pain (11.5±2.7), stiffness (5.3±1.3), and physical function (35.1±9.6) to "Injection 1" sub scores for pain (9.7±2.5), stiffness (4.2±1.5), and physical function (29.2±7.6) were recorded. These and the subsequent values listed below are represented by each of the consecutive data bars in Figure 1.

A decrease from "Injection 1" sub scores for pain (9.7±2.5), stiffness (4.2±1.5), and physical function (29.2±7.6) to "Injection 2" sub scores for pain (6.4±2.5), stiffness (3.0±1.3), and physical function (22.0±7.2) were recorded.

A decrease from "Injection 2" sub scores for pain (6.4±2.5), stiffness (3.0±1.3), and physical function (22.0±7.2) to "Injection 3" sub scores for pain (4.8±2.0), stiffness (2.2±1.1), and physical function (16.7±5.4) were recorded.

**Figure 2:**
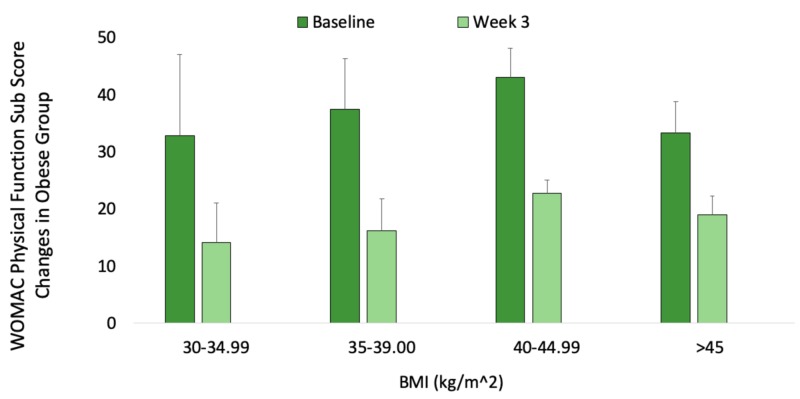
Mean reduction in Western Ontario and McMaster Universities Osteoarthritis Index (WOMAC) disability scores following Supartz FX injections into the knees of obese (BMI≥30.00 kg/m2) patients Weekly sub scores for physical function only were compared at baseline and at three weeks using BMI stratification of the obese group. Weekly percent changes are not included within this cohort. n=23.

Scores for the obese cohort (BMI ≥ 30.00 kg/m2) were analyzed by comparing baseline values of physical function only to those after the third injection. Neither mean percent improvement nor statistical significance were used to evaluate these specific changes within the obese group (Figure 2). 

**Figure 3:**
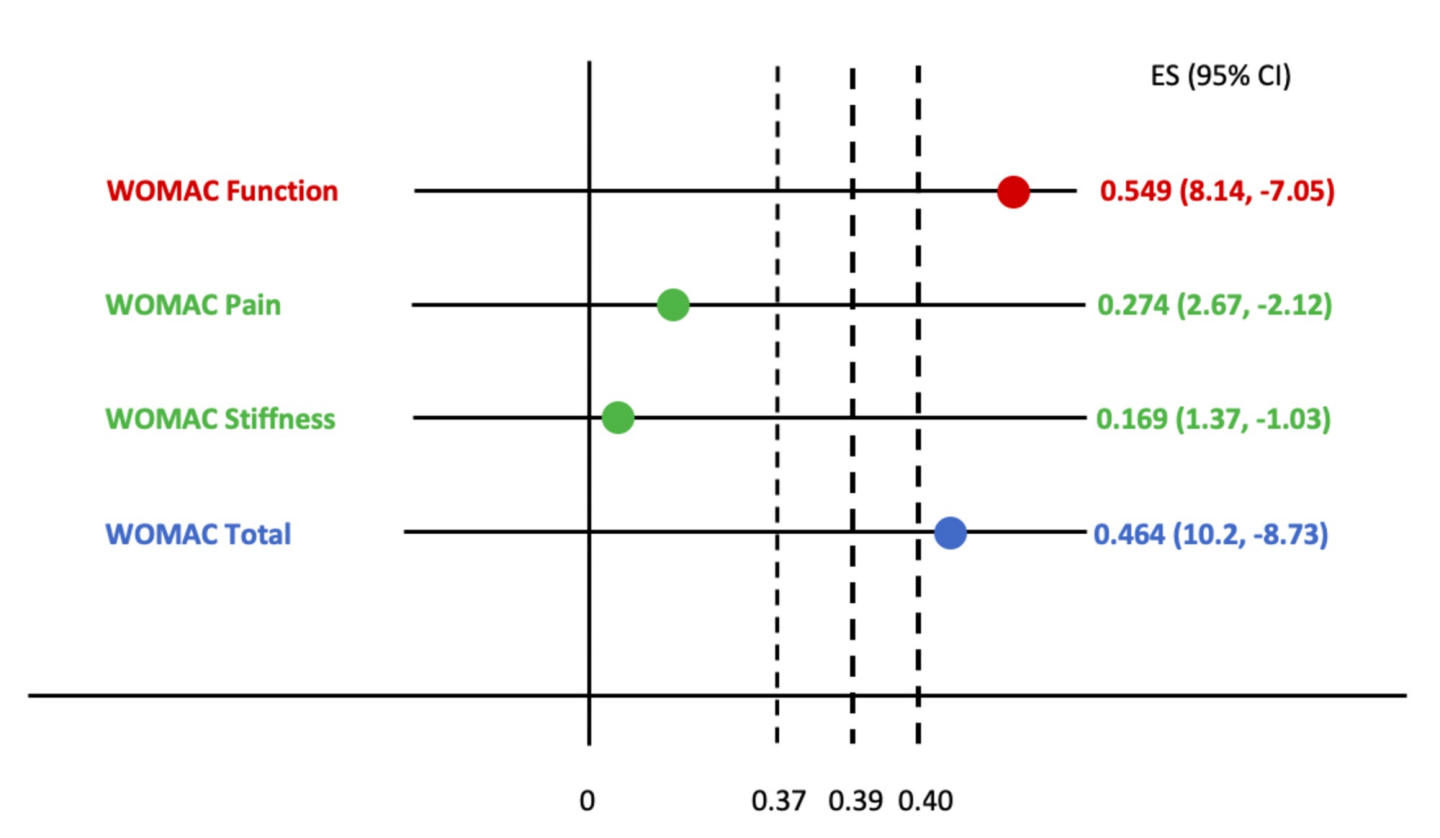
Confidence intervals of treatment effect sizes (ES) for Western Ontario and McMaster Universities Osteoarthritis Index (WOMAC) sub scores are compared to published minimum clinically important improvement (MCII) thresholds for pain (0.39), stiffness (0.39), function (0.37), and total outcomes (0.40) The MCII reflects the smallest clinical change that is considered important to patients.

Treatment effect sizes that were based upon weekly improvements in WOMAC sub scores are plotted against published MCII thresholds (Figure 3).

## Discussion

This study evaluates the clinical meaningfulness of changes in self-reported patient outcomes following hyaluronic acid injections into the osteoarthritic knee. We were unable to identify any previous studies that analyze self-reported outcomes following a short-term, three-week Supartz FX injection schedule. As such, the results of this study suggest the greatest mean improvement (%) occurs after just two injections, evidenced by the highest percent reduction in WOMAC scores occurs following the second injection (p<0.05) (Figure 1). The percent value indicated above each of the weekly mean sub scores corresponds to the percent reduction in pain, stiffness, and physical function; the larger the percent reduction, the greater the improvement in functionality.

Statistical significance in and of itself provides data regarding sample size but does not incorporate the procedure’s clinical significance to patients. Therefore, interpretation of results using MCII thresholds corroborates statistical significance by highlighting the smallest clinical change important to patients. Study results are considered clinically significant if the lower confidence interval of its treatment effect size is greater than the MCII, and not clinically significant if its upper confidence interval lies below or includes the MCII.

Variation may exist within different patient populations, causing discrepancies in what one patient considers clinically significant compared to another, either from subjective experience or baseline disease. However, the MCII serves as a reasonable proxy for evaluating meaningful improvement within populations of similar demographics. Thus, the MCIIs used here are derived from previously published data. In Guidelines for Treatment of Osteoarthritis of the Knee, the American Academy of Orthopaedic Surgeons (AAOS) used effect sizes reported by Angst et al. to compute thresholds for WOMAC sub scores of pain (0.39), function (0.37), stiffness (0.39), cumulative (0.40) [11].

Weekly mean sub score data shown in Figure 1 were analyzed for treatment effect size and compared to the above MCII thresholds (Figure 3). While statistically significant, these findings are only “possibly” clinically significant as the confidence intervals for each effect size include the MCII. Therefore, in concordance with the recommendations set forth by the AAOS, this study provides inconclusive evidence for short-term use of Supartz FX in the treatment or symptomatic management of knee osteoarthritis. While self-reported outcomes do show moderate improvement after two injections of Supartz FX, the changes are not considered clinically meaningful.

These results contribute to a discourse on osteoarthritis from an evidence-based medicine (EBM) perspective. EBM often incorporates the best evidence, clinical expertise, and patient wishes when making an informed decision. Therefore, while the evidence for use of IAHA is inconclusive (and not recommended by the AAOS), clinicians should exercise clinical judgment and accommodate patient preference when explaining the benefits and harms of IAHA. Certain groups of patients may respond better to intra-articular Supartz FX than others. For example, Figure 2 highlights decreasing self-reported WOMAC scores for patients of different BMI groups. Though not directly linear, a physician who believes in the efficacy of hyaluronic acid may be more likely to combine weight-loss efforts with injections for obese patients, to achieve potentially greater overall self-reported outcomes.

## Conclusions

The use of intra-articular hyaluronic acid injections has been well studied, though evidence of improvement in pain, stiffness, and disability versus placebo has been controversial. Further data supporting its efficacy has been unconvincing, and the AAOS does not recommend the use of IAHA in clinical practice. Despite manufacturer recommendations, short-term use of Supartz FX for knee osteoarthritis does not meet clinically significant thresholds as the treatment effects for WOMAC sub scores fail to satisfy published MCII criteria.
